# Usability and Evaluation of a Health Information System in the Emergency Department: Mixed Methods Study

**DOI:** 10.2196/48445

**Published:** 2024-02-21

**Authors:** Christina Østervang, Charlotte Myhre Jensen, Elisabeth Coyne, Karin B Dieperink, Annmarie Lassen

**Affiliations:** 1 Department of Emergency Medicine, Odense University Hospital Odense Denmark; 2 Department of Clinical Research, University of Southern Denmark Odense Denmark; 3 Department of Orthopedic Surgery and Traumatology, Odense University Hospital Odense Denmark; 4 School of Nursing and Midwifery Griffith University Brisbane Australia; 5 Department of Oncology, Odense University Hospital Odense Denmark; 6 Family Focused Healthcare Research Center (FACE), University of Southern Denmark Odense Denmark

**Keywords:** consumer, eHealth, elderly, emergency department, emergency, family members, healthcare professionals, information system, mixed methods research: patients, qualitative interview, questionnaire, technology, usability, usable

## Abstract

**Background:**

A lack of information during an emergency visit leads to the experience of powerlessness for patients and their family members, who may also feel unprepared to cope with acute symptoms. The ever-changing nature and fast-paced workflow in the emergency department (ED) often affect how health care professionals can tailor information and communication to the needs of the patient.

**Objective:**

This study aimed to evaluate the usability and experience of a newly developed information system. The system was developed together with patients and their family members to help provide the information needed in the ED.

**Methods:**

We conducted a mixed methods study consisting of quantitative data obtained from the System Usability Scale questionnaire and qualitative interview data obtained from purposively selected participants included in the quantitative part of the study.

**Results:**

A total of 106 patients and 14 family members (N=120) answered the questionnaire. A total of 10 patients and 3 family members participated in the interviews. Based on the System Usability Scale score, the information system was rated close to excellent, with a mean score of 83.6 (SD 12.8). Most of the participants found the information system easy to use and would like to use it again. The participants reported that the system helped them feel in control, and the information was useful. Simplifications were needed to improve the user experience for the older individuals.

**Conclusions:**

This study demonstrates that the usability of the information system is rated close to excellent. It was perceived to be useful as it enabled understanding and predictability of the patient’s trajectory in the ED. Areas for improvement include making the system more usable by older individuals. The study provides an example of how a technological solution can be used to diminish the information gap in an ED context.

## Introduction

### Background

Clear communication and information are essential to improving care and patient outcomes in the emergency department (ED) [1-5]. A lack of information during ED visits causes patients and their family members to experience a sense of powerlessness and to feel unprepared to cope with acute symptoms [2,3,6]. Due to the hectic nature of the ED and the constant interruptions, communication from health care professionals is often inadequate or not tailored to patients and their families [4,7]. While this problem has been known for many years, it still persists to this date [1].

Health technologies are implemented in many parts of health care systems to promote quality care and treatment [8]. The design and purpose of health technologies range widely from organizational [9] to person-centered intentions [10]. In the ED, technologies may be used as quality dashboards [9] and more personal information systems on patients’ own devices to support the delivery of health information [11]. However, the successful use of technology in clinical practice is likely to be ineffective if user needs are not carefully addressed and incorporated before attempting a full-scale implementation [9,12]. Thoroughness in integrating and understanding user perspectives will have a direct impact on how well the technology is suited for clinical practice [13,14].

Based on the current findings, patients in the ED and their family members have unmet information needs [1-4]. Hence, guided by the principles of user-driven activities [15], a health information system was developed [16]. The health information system, which is called “Cetrea Clinical Logistic (CCL) *for patients*,” is available for patients in the hospital’s emergency room and displays real-time information, including (1) person-centered activities, (2) information videos, (3) a notepad, (4) waiting time, and (5) the nurse and physician responsible for care.

Usability is one of the factors affecting the acceptance of health information systems by users, and it is essential for the effective use of the system [17]. A usability evaluation can identify problems and weaknesses in the design and functionalities in the early development phase [18]. Usability tests allow developers to address and adjust concerns and, thus, avoid implementing technologies that will not be useful in the clinical context.

Therefore, a usability evaluation from an end-user perspective was completed to obtain a nuanced understanding of the sustainable use of the system, specifically from the perspective of patients and their family members.

### Objective

The objective of this study is to gain knowledge about the usability and experiences of the newly developed information system, CCL for patients. This study reports on patients’ and family members’ evaluations of this system.

### Participatory Design and Technology

**Figure 1:**
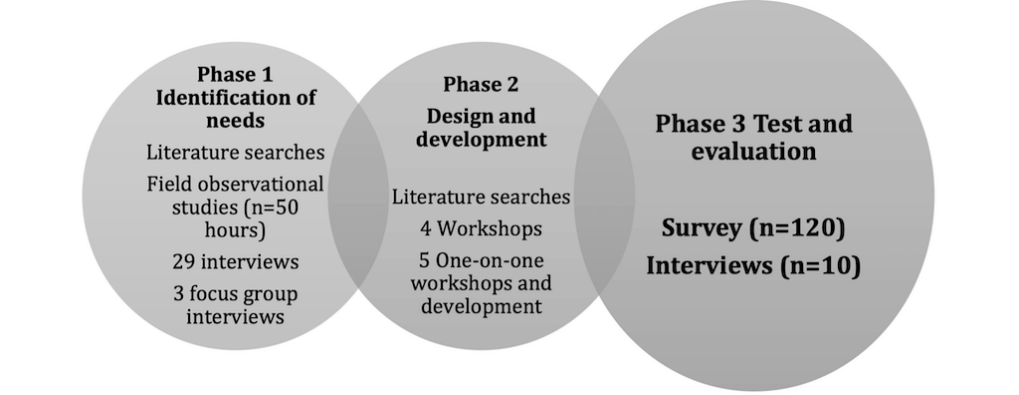
Overview of the 3-phase study, highlighting the evaluation phase, which is reported in this study.

This study is the final phase of a 3-phase participatory design study (Figure 1) [19]. Participatory design is a research methodology based on the epistemological position of genuine involvement and understanding of the needs of future end users. A new technology can be designed to improve a real-life problem [20]. The core principles in participatory design methodology have been the theoretical framework of the overall study. In the initial phase, the author group identified the essential needs of patients in the ED, their family members, and ED clinicians [2,3]. The results from phase 1 informed the second phase, in which an information system, CCL for patients, was developed in a cocreation process [16]. The third phase involved testing and evaluation of the system, which is reported in this study. Reporting the evaluation of participatory-designed health technology is a common part of the research methodology [21,22].

The author group has had no financial interest in the system owners of CCL for patients and has no interest in either marketing or promoting the system.

CCL for patients provides information directly to patients and their family members during their stay in the ED. The information provided relates to treatment and time factors and is adjusted toward the individual patient. CCL is an already existing and implemented system for task management for clinicians’ use only [23], whereas CCL for patients is a redesign and further development of the system for patients’ use.

**Figure 2:**
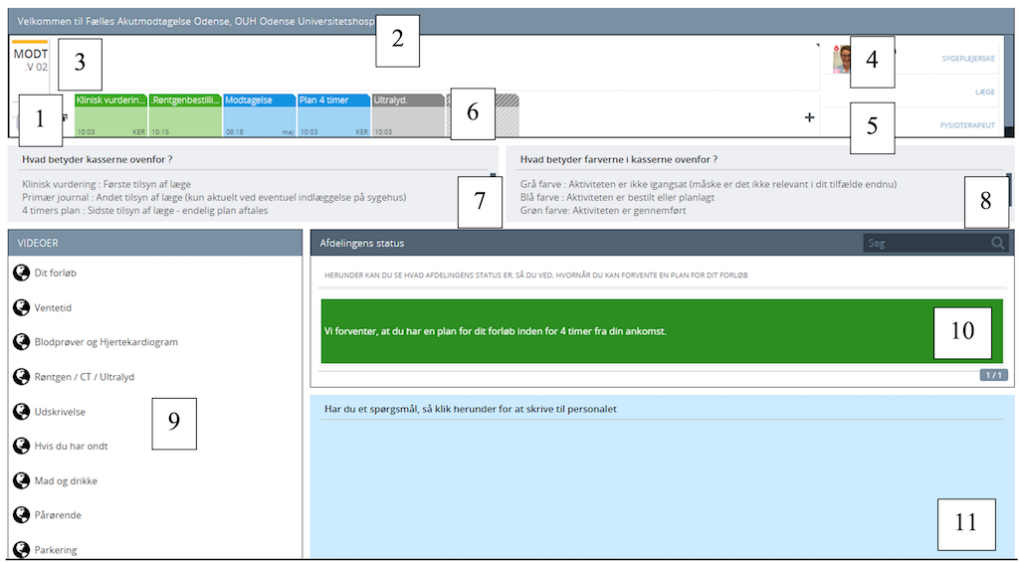
Cetrea Clinical Logistics (CCL) for patients and its functionalities, as displayed to patients and their family members, developed in the second phase of the overall study (the figure has been previously published by Østervang et al [16]). (1) Number of the ED room. (2) Name of the hospital department. (3) The name of the patient (no sensitive information is displayed). (4) The nurse who is responsible for care. (5) The physician who is responsible for treatment. (6) Process line with activities. Displaying nurse assessments, blood samples, electrocardiograms, physician assessments, X-rays, etc. (7) Clarification of the different colors in the process line. Gray: not started; blue: activity scheduled; and green: activity finished. (8) Clarification of special activity names. (9) Link to information videos (eg, information on discharge). (10) Three diverse colors indicate the estimated waiting time: less than 4 hours, equal to 4 hours, or more than 4 hours, respectively. (11) The shared note pad for the patients to write questions to health care professionals or messages from family members.

The functionalities of the CCL for patients’ screen are presented in Figure 2.

## Methods

### Research Design

**Figure 3:**
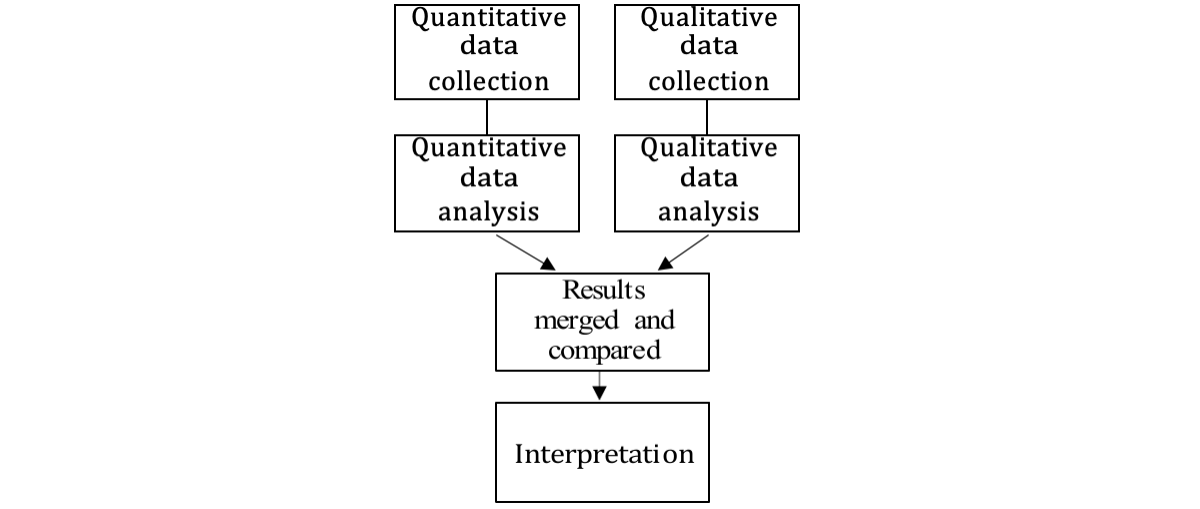
Diagram for a study using convergent design (Creswell and Clark) [24].

This is a mixed methods study inspired by a convergent parallel design [24]. This design was chosen to obtain nuanced insights into the usability of the system. Further, we adopted this approach to usability testing because quantitative data can identify usability issues and dissatisfaction with program design, while qualitative data can provide detailed information about the causes of the usability issues and point at potential methods for program optimization. As shown in Figure 3 [24], the study contained the following two parts, ending with a merged result: (1) a questionnaire and descriptive characteristics of the participants, and (2) semistructured interviews with patients and their family members.

### Setting

The data were collected in Odense University Hospital’s ED between August 22 and September 29, 2022, on weekdays from 8 AM to 5 PM. The information system was displayed on a laptop personal computer (PC) sitting on the bedside table in the ED room. Four PCs were used during the test phase. They were installed in the specific ED room where the patients participating in the study were admitted.

### Inclusion Criteria and Recruitment

All patients admitted to the medical area of the ED without a final plan for treatment and care were eligible for participation. Patients were excluded if they were severely ill or cognitively unable to use the technology. However, patients who were excluded due to a cognitive inability to use the screen but who were still able to give consent for their family members’ participation were enrolled if the family member was interested in participating. Patients were recruited by the first author (CØ) or one of 2 research assistants, all of whom have a Master of Nursing Science degree and research experience. Potential participants were identified and discussed with the responsible care nurse before they were approached to reduce the possibility of any concerns.

### Quantitative Phase

A survey was conducted to elicit the opinions and experiences of patients and their family members using the information system.

#### The Questionnaire

The questionnaire, the System Usability Scale (SUS), contained questions regarding the usability of the system. Answers are rated on a 5-point Likert scale from “strongly disagree” to “strongly agree,” with 5 representing the highest score (strongly agree) [25]. The participants answered 10 questions from the SUS and 2 questions specific to this study (questions 11 and 12) [25]. These 2 extra questions were added to obtain general information about the participants’ experience with CCL for patients (question 11: “I think the system provided a great overview of my stay,” and question 12: “I think the information in the system made sense to me”). As SUS has been translated and validated in a Danish hospital context previously (Cronbach α=.87) [26], it was considered suitable for this study.

#### Sample Size

A total SUS score between 70 and 90 indicates good to excellent usability of the tested system [27]. Based on previous research conducted in Scandinavia using SUS in health care with a reported mean score of 79.81 (SD 14.28), we would gain a 95% CI for a mean score between 77.2 and 82.4 if a total of 120 patients were included [28].

#### Data Collection

If a patient agreed to participate, the researcher cooperated with the local IT department at the hospital to ensure the patient’s access to the system. Initially, the researcher sent the IT department an SMS text message providing information on the PC number and the ED room number. The IT specialist matched the PC and room numbers. Then, the researcher double-checked that the correct information was displayed before handing it to the patient. All participants were given oral guidance on how to use CCL for patients.

The PC with individual information was placed on the bedside table until either the patient left the ED, the patient had used the system for a minimum of 2 hours, or the patient felt ready to perform the evaluation. All of this had to happen no later than 5 PM, when the IT department closed. When returning the PC, the participants were given an iPad to fill out the questionnaire. The data were stored on the logged server OPEN [29], which is part of Odense University Hospital and the University of Southern Denmark.

### Qualitative Phase

Interviews were conducted with individual patients or with the patient together with a family member to get a deeper insight into their experiences using the information system.

#### Interviews

The qualitative part included a subset of the participants from the quantitative part. Before making CCL for patients available to the participants, they were asked whether they were interested in participating in an interview.

All interviews were conducted by the first author (CØ). By taking a phenomenological-hermeneutical stance, CØ was allowed to recognize her perceptions as an experienced emergency nurse within hermeneutic interpretation [30]. To bridle her preconceived ideas, CØ wrote down her preunderstandings of why patients lack information in the ED. This reflection provided an initial focus for both the overall research question and the interview questions.

The interviews were conducted in the hospital room after the participants had completed the questionnaire. Notes and quotes were taken during the interview. A summary of the conversation was generated at the end of the interview in the form of member checking [31]. A semistructured interview guide inspired by Kvale was used [32]. An example of a question is: “What was your experience of using CCL for patients?” The interviews lasted up to 30 minutes. The interviews were conducted until no new themes arose [33].

#### Sample Size

To obtain maximal variation, a purposive sampling strategy was used [33]. The inclusion criteria were the same as for the quantitative part of the study, but they also ensured representation of differences in age and gender.

### Analysis

#### Analysis of the Questionnaires

Only fully completed questionnaires were analyzed (N=120). There were no missing data, as the questionnaire was only considered complete if all the questions were answered. According to the SUS guidelines, we performed an individual analysis of each participant’s SUS score as well as the mean value for the entire population. We separated the 2 self-constructed questions from the original SUS questions in the calculation and interpretation process to ensure that they were accurate and reliable. The final score was between 0 and 100, where a higher score indicates better usability. Odd-numbered questions were positive in tone, and even-numbered questions were negative in tone, so the scale was converted into points ranging from 1 to 5 (1=strongly disagree to 5=strongly agree). The final score was calculated as follows: X = the sum of the points for all odd-numbered questions minus 5. And Y = 25 minus the sum of the points for all even-numbered questions. SUS score = (X + Y) × 2.5 [34]. A system needs a score above 70 to be considered acceptable; better systems will score from the high 70s to the high 80s, and excellent systems will score above 90 [27].

#### Analysis of the Interviews

The qualitative interviews were analyzed and reported based on Malterud’s [35] systematic text condensation. This process consisted of four steps: (1) transcriptions were read several times to get a total impression of the data and to find preliminary themes; (2) we identified and sorted meaning units based on the preliminary themes and arranged them into code groups; (3) the code groups were reviewed, and the content was reduced into condensates; and (4) the meaning and content of the condensates were synthesized and interpreted [35]. The analysis was completed by CØ using NVivo (version 12; QSR International). The trustworthiness and rigor of the qualitative part of the study were evaluated using Guba’s [36] definition of quality criteria. As part of steps 2, 3, and 4 in the analysis, the emerging themes and codes were discussed in the author group toward strengthening the credibility and reflexivity of our interpretation of the interviews. Using a systematic approach toward the analysis strategy of all interviews ensured confirmability in the data collection and analysis process.

The SQUIRE 2.0 checklist [37] was used to create transparency and ensure that no important information was missed in the reporting of the study.

#### Integration of Quantitative and Qualitative Results

To achieve an expanded understanding of the results, the qualitative and quantitative results were compared and integrated as the final step of the analysis using joint display tables [24]. In a joint display table, the 2 results are presented in a way that allows comparison, leading to confirmation, disconfirmation, or expansion of each other [24]. The results from the SUS (quantitative results) are presented on a Likert scale, showing the variation of the grades in the different questions. To elaborate on and verify the answers, supportive qualitative quotes were presented for each question. We divided the grades into low (1-3) and high (4-5) to separate the different perceptions of CCL for patients.

### Ethical Considerations

All the participants received verbal and written information about the study in accordance with applicable ethical rules [38] and provided their oral and written consent. The study is registered with the Danish Data Protection Agency, Fortegnelsen (19/22672). Approval of the project was granted by the Regional Committee on Health Research Ethics for Southern Denmark (S-20192000–111).

## Results

### Quantitative Results

In total, 14 family members and 106 patients agreed to participate. A total of 27 patients declined to participate for three main reasons: (1) no interest, (2) no technical skills, and (3) a lack of mental ability due to the acute situation.

**Table 1:** Demographic descriptions of the participants.

Demographic description			Patients (n=106)		Family members (n=14)		Total (N=120)
**Gender, n (%)**							
	Female	55 (51.9)		8 (57.1)		63 (52.5)	
	Male	51 (48.1)		6 (42.9)		57 (47.5)	
Age (years), mean (SD)			55.5 (SD 18.7)		66.5 (SD 11.6)		57 (SD 18.3)
**Civil status, n (%)**							
	No partner	39 (36.8)		2 (14.3)		41 (34.2)	
	In a relationship	67 (63.2)		12 (85.7)		79 (65.8)	
**Children, n (%)**							
	Having children	81 (76.4)		14 (100.0)		95 (79.2)	
	Having children living at home	32 (39.5)		6 (42.9)		38 (40.0)	
**Technology, n (%)**							
	Having a smartphone	96 (90.6)		14 (100.0)		110 (91.7)	
	Using technology on daily basis	102 (96.2)		13 (92.9)		115 (95.8)	
**Education, n (%)**							
	Low	21 (19.8)		1 (7.1)		22 (18.3)	
	Medium	71 (67.0)		9 (64.3)		80 (66.7)	
	High	14 (13.2)		4 (28.6)		18 (15.0)	

The respondents were equally represented by gender, with a mean age of 57 years. The mean age of family members was higher than that of the included patients. Most participants had medium education levels, but low and high educational levels were also represented.

Overall, the participants answered the survey positively. As displayed in Tables 2 and 3, each item could have a score contribution between 1 and 5. All the odd-numbered (positive) questions had a score contribution above 4.27-4.53, and all the even-numbered (negative) questions had a score ranging from 1.52 to 1.99. Question 1 had the most positive answers: 94.2% (113/120) strongly agreed or agreed that they would like to use the system if they were hospitalized again. Question 4 had the highest negative score value, indicating that the participants felt they needed help using the system. Of the participants, 50.8% (61/120) indicated that they were confident using the system, answering “strongly agree” to question 9, and 87.5% (105/120) strongly agreed or agreed that most people would be able to learn to use this system.

**Table 2:** Results of the System Usability Scale for all participants (N=120) and the System Usability Scale score contribution of individual items.

System Usability Scale analysis item	Value per 5-point Likert scale response, n (%)						Score contribution (1-5), mean (SD)
	1 (strongly disagree)	2 (disagree)	3 (neutral)	4 (agree)	5 (strongly agree)		
1. I think I would like to use this system, if I am admitted again.	0 (0)	0 (0)	7 (5.8)	42 (35)	71 (59.2)	4.53 (SD 0.61)	
2. I found the system unnecessarily complex.	61 (50.8)	41 (34.2)	11 (9.2)	5 (4.2)	2 (1.7)	1.72 (SD 0.92)	
3. I thought the system was easy to use.	1 (0.8)	1 (0.8)	8 (6.7)	39 (32.5)	71 (59.2)	4.48 (SD 0.73)	
4. I think that I would need help from the staff to be able to use this system.	49 (40.8)	44 (36.7)	11 (9.2)	11 (9.2)	5 (4.2)	1.99 (SD 1.12)	
5. I found the various functions in the system to be well correlated.	1 (0.8)	1 (0.8)	8 (6.7)	65 (54.2)	45 (37.5)	4.27 (SD 0.69)	
6. I thought there was too much inconsistency in this system.	56 (46.7)	52 (43.3)	8 (6.7)	1 (0.8)	3 (2.5)	1.69 (SD 0.84)	
7. I would imagine that most people would learn to use this system very quickly.	0 (0)	0 (0)	15 (12.5)	53 (44.2)	52 (43.3)	4.31 (SD 0.68)	
8. I found the system very cumbersome to use.	70 (58.3)	42 (35)	5 (4.2)	2 (1.7)	1 (0.8)	1.52 (SD 0.73)	
9. I felt very confident using the system.	2 (1.7)	6 (5)	4 (3.3)	47 (39.2)	61 (50.8)	4.33 (SD 0.89)	
10. I needed to learn a lot things before I could get going with this system.	67 (55.8)	42 (35)	7 (5.8)	4 (3.3)	0 (0)	1.57 (SD 0.75)	

Based on the answers to the 2 self-constructed questions, (Table 3), 57.5% (69/120) of the participants strongly agreed that CCL for patients provided a great overview of their stay, and 87.5% (105/120) agreed or strongly agreed that the information in the system made sense to them.

**Table 3:** Results of general questions calculated by System Usability Scale principles for all participants (N=120) and the System Usability Scale score contribution of individual items.

System Usability Scale analysis item	Value per 5-point Likert scale response, n (%)						Score contribution (1–5), mean (SD)
	1 (strongly disagree)	2 (disagree)	3 (neutral)	4 (agree)	5 (strongly agree)		
11. I think the system provided a great overview of my stay.	0 (0)	2 (1.7)	10 (8.3)	39 (32.5)	69 (57.5)	4.46 (SD 0.72)	
12. I think the information in the system made sense to me.	1 (0.8)	5 (4.2)	9 (7.5)	45 (37.5)	60 (50)	4.32 (SD 0.85)	

For all participants, the total mean score for the SUS scale was 83.6 (SD 12.8), indicating that the system had close to excellent usability.

**Figure 4:**
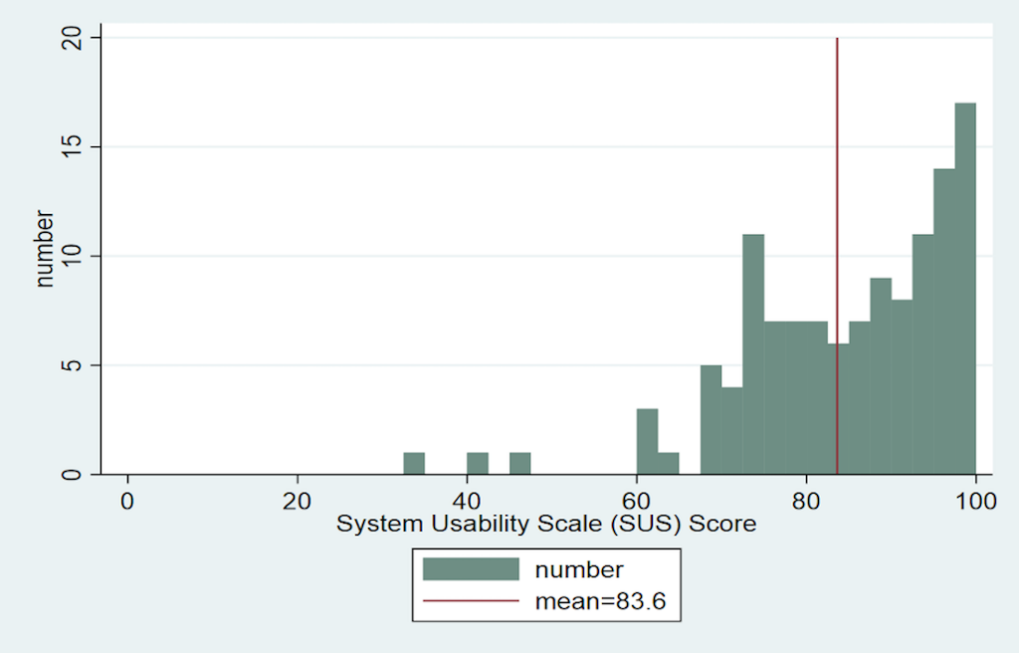
Overview of the System Usability Scale (SUS) rating table with inserted value ranges [27].

The median score was 85, and Figure 4 [27] shows the distribution of the individual answers. The scores covered the entire range from 0 to 20 persons per score, and the majority of individuals scored above 70.

### Qualitative Results

A total of 10 patients and 3 family members (1 daughter aged 55 years, 1 son aged 65 years, and a husband aged 37 years) were interested in elaborating on their experience of CCL for patients after they had tested the system, and the questionnaire was completed. The patients were aged between 32 and 96 years, with equal representation of men and women, and 3 patients were retired.

The following three main themes emerged from the analysis: (1) future perspectives on usability and design; (2) means toward empowerment; and (3) family implications. These themes will be elaborated on using quotes in the upcoming sections.

### Future Perspectives on Usability and Design

The majority of the participants expressed a very positive attitude toward CCL for patients but also offered ideas for the future design of the system. The part of CCL for patients that displayed the estimated waiting time in the ED was found to be intuitive and easy to understand and provided informative insights that prepared the participants for their length of stay. This reduced their frustration with not knowing. However, they expressed concerns about the system’s lack of familiarity and that it could be improved if the design was like other systems they used in everyday life, such as email or smartphone apps.

The system was not difficult at all but I think it would benefit from more recognizability with others systems, for example, email or iPhone applications.

Male in his 60s

Most participants valued the line that displayed the boxes with activities the most. They found this part of the system to be essential, as it was the only part that provided direct, personalized information. While they all expressed that they were able to understand the meaning of the changing colors, they also suggested that the text in the boxes could be provided in plain language or a “help” function with text or video could be used to explain the activity in the box.

The line with the boxes could be much larger, as this is the most important part! It would be great if you could choose whether you would like to see only the line or all actions on the screen.

Joint interview, male patient in his 80s and daughter in her 50s

The participants all watched more than one video, and there was a consensus that the content in the videos was helpful. A few patients who were placed in the hallway due to crowding found it difficult to listen to the videos not only because of the general noise but also because they were afraid of disturbing others. However, the information provided by using videos instead of text was appreciated.

The content in the videos was exactly the information I needed. It was nice to be able to revisit the information in the video.

Female patient in her 50s

A participant found CCL for patients to be too general. More personalized information, such as individual test results, should be incorporated. Moreover, patients who were visually impaired found the system difficult to use.

I have difficulties with my vision, and I do not think I would have been able to use this without help.

Female patient in her 60s

### Means Toward Empowerment

All of the participants agreed that the system provided an overview that otherwise would not have been accessible for them. Knowing who their treating nurse and doctor were calmed the participants. They described a feeling of not being forgotten in the hectic environment of the ED. Moreover, they valued being able to follow when activities changed from passive to active. Consistency between actions on the screen and in real life provided them with confidence in health care professionals.

When you are here, you can hear people working, but you do not know if anyone is taking care of your situation, or you are forgotten. The system helped us to believe we were not forgotten (…) We loved that when something happens on the screen then it was also reflected in real life. E.g. when the screen said the doctor was on his way- he actually came.

Joint interview, female patient, and husband in their 30s

Several of the participants stated that having CCL for patients available made them feel calm, as the system provided predictability. Further, having an overview helped them to remain in control of the course of treatment in the ED. Some of the participants said this system could save the nurses’ time, as they felt they were more empowered to handle the situation in the ED since they knew what they were waiting for.

The questions that I would have needed a nurse to answer were provided by the system; that was really great.

Female patient in her 30s

A few of the participants were worried that CCL for patients would need resources from health care professionals that were already scarce.

I am worried that the system takes time away from the patients to support the system.

Joint interview, male patient in his 80s and daughter in her 50s

### Family Implications

Both patients and family members indicated that giving family members direct access was important. CCL for patients gave the family up-to-date information about the care and treatment-related interventions as soon as they attended the hospital room, and they did not need to wait for a nurse or doctor to get an idea of what was planned.

My mother is not able to remember what she is planned for today. I think it was great for me to see she is waiting for X rays.

Female patient in her 90s and son in his 60s

Furthermore, the family members reported that the system helped them to support the patient, as they could keep track of the interventions provided by CCL for patients. Family members of older patients felt the system was too complicated for the older individuals to use but appreciated that the system was available for them because it allowed them to talk the patient through the stay in the ED. Moreover, a family member stated that the system made it possible for him to let his wife go to sleep, as they agreed that he would wake her up when he saw that activities were about to happen.

It was nice for me to have a system that told us when things were going to happen. My wife fell asleep, and I knew I did not need to wake her up before I could see the box turned into the blue color. It was easy to understand.

Joint interview, female patient, and her husband in their 30s

### Merged Data

We combined the quantitative and qualitative data in a joint display (Multimedia Appendix 1), providing an assessment of the quantitative and qualitative data together. In this way, the data allow us to expand our understanding of patients and their family members’ experiences with CCL for patients. For example, in question 1, the participants were asked whether they would like to use CCL for patients again. The participants who gave a lower score (1-3) to that question were concerned if the system would replace personal appearance from health care professionals, whereas those who gave it a high score (4-5) valued how the systems helped them to keep control.

Furthermore, question 7 regarding people’s ability to learn to use the system revealed that the participants who gave a low score (1-3) wanted more simplicity, fearing that the older patients would find the system difficult. Meanwhile, the participants who gave high scores (4-5) felt that the system was easy to use. Regarding question 11, the majority of the patients and their family members stated that CCL for patients provided a great overview of the patient’s pathway. They further elaborated on this in the interviews, as they felt that the overview of care in the system helped them to feel less stressed and better understand the treatment pathways.

## Discussion

### Principal Findings

In this study, we report that the perceived overall usability of the health information system CLL *for patients* is good to excellent, providing information that is needed during the entire emergency process. The participants rated the system highly (a score of 83.6 points) and reported that the system gave them an opportunity to remain in control, as they knew what they were waiting for and who was responsible for care and treatment.

### Technology as a Means to Empower Patients and Family Members in the ED

Looking into previous research on testing systems using SUS [28], a mean score of 83.6, as found in this study, would indicate that the tested system was successful. However, while CCL for patients was evaluated positively overall, we also uncovered technical concerns regarding usability limitations, specifically regarding the older individuals. Our results showed a mean patient age of 57 years, which represents a relatively young ED population. However, the mean age of the family members was almost 10 years (9.5 years) older. The older individuals found the system to be complicated to use and felt that it needed simplified functions, such as a zoom function and recognizability (eg, other well-known systems). Echoing these findings, Verma et al [39] investigated the level of eHealth literacy among older adults and caregivers and found that one main barrier to the adoption of eHealth was a lack of familiarity with the tools available. In the development phase of CCL for patients [16], decisions had to be made for the system to work in a clinical setting. One decision was the use of an interface design, which did not allow us to integrate well-known functions, for example, from email or application symbols. Our results highlighted that it might not be possible to design technologies using a one-size-fits-all approach. However, in line with previous research [40], we discovered that the usability testing allowed the developers to adjust and isolate functionalities to provide improved usability outcomes in the future. For example, we found that the participants valued the display with the boxes, which could be promoted in a revised version by the availability of a zoom function.

Furthermore, the participants expressed concerns about whether CCL for patients would influence the health care professionals’ available time to provide actual care. Barriers to the adoption of technology systems in clinical settings include the workflow or demand for more human resources [12]. As the information system is a redesigned patient flow system, it would not require changes in workflow or unduly burden professional health care resources. Another consideration was the need for personal test results. They could not be provided in the current form of the system, as it would require a personal log-on to avoid safety issues related to General Data Protection Regulations.

The participants who rated the usability the highest explained that the system made them feel that they were in control of the situation without the fear of being forgotten. The system provided an overview of the care transition and, therefore, offered predictability. This need to be in control has been identified in another study, which described patients’ and their relatives’ dissatisfaction when visiting the ED [6], as they felt powerless in the ED. Not having knowledge or information available led to such feelings of powerlessness. Nursing rounds were suggested in that study to improve information support [6]. Our results showed that the patients felt more independent because they were able to find the needed information using technology.

Being acutely ill places individuals in a vulnerable situation, and their cognitive capabilities are challenged [2]. Communication from health care professionals and how information is presented have a significant influence on how that information is comprehended [2,41,42]. In this study, we developed information videos related to the journey within the ED, and the participants reported that they were an accessible and usable way to understand information in a stressful situation. Patients and their family members declared that this gave them a feeling of empowerment. Indeed, empowering patients to be in control and involved in their own care is recognized as a core value of high-quality patient-centered care [43]. As Emmamally et al [44-46] noted, improved partnering with family members in the ED is needed. If the family is not included, there is an increased risk of miscommunication and poor understanding of health-related matters [2,44,47-49]. However, creating a closer partnership of care has been described as challenging within the ED due to the high workload, overcrowding, and multitasking [47]. This is echoed in recent findings from studies conducted in a Danish context [2,3], in which family members requested more systematic inclusion in the ED. In this study, the results showed that CCL for patients was perceived as usable and as a useful way to systematically include families during the ED stay.

An update of the Medical Research Council’s guidelines for developing and evaluating complex interventions in health care states that appropriate users should be involved in every part of the development, process, and outcome analysis of a complex intervention to ensure sustainable interventions [50]. In line with best practices, the information system has been developed together with representatives of future users of the system, including health care professionals, managers, patients, family members, and IT specialists [16]. For decades, the ED context has been a hectic environment [4,42,51,52]. This creates challenges at both the information and communication levels, affecting whether patients and their families feel in better control during their stay in the ED [1,4,42,51,52]. In this study, we presented and evaluated a simple but unique system that provides timely information to empower individuals without straining health care professionals’ resources. The usability test was a crucial and important step to inform changes in functionalities and experiences of using IT in the ED.

### Strengths and Limitations

Questionnaires are a common and recognized method for evaluating the usability of health technologies. However, the contextual factors affecting the results are difficult to determine [53]. The SUS did not provide insights on the effectiveness or efficiency of the system, but it is a validated questionnaire and provided an overall understanding of the system [27]. The mixed methods approach [24] enabled the integration of quantitative and qualitative data. This allowed us to obtain an understanding of how the usability was rated and why the results emerged for the specific questions, which is considered a strength of usability testing [40,54].

Additionally, our findings serve as an inspiration to others about how a participatory design process can develop a technology that is aligned with some of the essential needs described by the users of the ED. The findings provide an example of how a technological solution can be used to reduce the information gap in an ED context, as the provision of adequate information to patients and their families is found to be a major challenge in an ED context [2,4,42].

This study also had some limitations. Using a broader evaluation method, for example, a qualitative evaluation questionnaire or an evaluation instrument with more domains, could potentially have provided the study with more nuances [55]. Patients attending the ED outside of the IT department’s business hours were not able to use the system. Therefore, we do not know if patients attending the ED in the late evening hours or at night would rate the usability differently. Moreover, no cognitive debriefings or adjustments were made specifically for individuals attending an ED, as these tests were conducted before introducing the questionnaire. Multimedia Appendix 2 [26,56] contains further details about the process as well as final modifications to the questionnaire. In addition, our results are based on a relatively young population (with a mean age of 57 years). Another weakness is that we did not include all users in the evaluation phase, as health care professionals, IT specialists, and managers were only involved in the development phase and not in the usability testing. For the system to be fully useful, it must run on its own or be serviced directly in the ED. These aspects will be considered in the planning of a future implementation process. Moreover, the transferability of the results is limited to countries with comparable access to and understanding of technologies, as in the Danish population and health care system.

### Conclusion

Based on the results of this study, the usability of CCL for patients is rated close to excellent by patients and family members. CCL for patients was perceived to be useful, as it enabled understanding of the ED treatment and pathway. The patients indicated that they, from the technology, were able to understand what was going to happen, experienced the feeling of being in control, and found the information to be useful. Areas for improvement include making the system more usable for the older individuals. It is concluded that a technological solution can be used to minimize the information gap in an ED context from the perspective of patients and their family members.

## Appendix

Multimedia Appendix 1 Joint display.

Multimedia Appendix 2 Supplementary file for the Methods section.

## Abbreviations

ED: emergency department
PC: personal computer
SUS: System Usability Scale
