# Supplementary material for: Usability and Evaluation of a Health Information System in the Emergency Department: Mixed Methods Study
Source: JMIR Hum Factors. 2024 Feb 21;11:e48445. doi: 10.2196/48445 (PMC10918535; doi:10.2196/48445)
Supplement: Multimedia Appendix 1 [file humanfactors_v11i1e48445_app1.pdf]

	Quantitative results (n=120)					Qualitative results (n=10)	
	**Strongly disagree 1 n (%)**	**Disagree 2 n (%)**	**Neutral 3 n (%)**	**Agree 4 n (%)**	**Strongly agree 5 n (%)**	**Answering scale 1–3 Themes** *Selected quotes*	**Answering scale 4–5 Themes** *Selected quotes*
Q1. I think I would like to use this system, if I am admitted again.	0 (0.0)	0 (0.0)	7 (5.8)	42 (35.0)	71 (59.2)	Means towards empowerment	
						I am worried that the system takes time away from the patients to support the system.	*I think this system was very good. The boxes with activities made me feel in control of what happened. I really liked that.*
Q2. I found the system unnecessarily complex.	61 (50.8)	41 (34.2)	11 (9.2)	5 (4.2)	2 (1.7)	Future perspectives on usability and design	
						It was a bit difficult, mostly because the text in the boxes was not provided in a plain language but in clinical terms.	*The system was not difficult at all, but I think it would benefit from more recognizability with other systems, for example, email or iPhone applications.*
Q3. I thought the system was easy to use.	1 (0.8)	1 (0.8)	8 (6.7)	39 (32.5)	71 (59.2)	Means towards empowerment	
						I like the boxes but did not dare to touch the screen to start the videos. No…	*Everything about this is easy. I am a big fan already. If I come to the hospital, I would like to have the system again, as it made me calm and in control.*
Q4. I think that I would need help from the staff to be able to use this system.	49 (40.8)	44 (36.7)	11 (9.2)	11 (9.2)	5 (4.2)	Future perspectives on usability and design	
						I think the system should start with an introduction film. I missed that. I think the use of colors should be considered more…use contrast colors that would be easier for me to read.	*I think it is very easy to use! It was nice you gave an introduction, because I was a bit stressed in the situation. But when all the stress was over, I really loved having the system to guide me through.*
Q5. I found the various functions in the system to be well correlated.	1 (0.8)	1 (0.8)	8 (6.7)	65 (54.2)	45 (37.5)	Future perspectives on usability and design	
						You need to work on the design. The boxes need to be large. I would like a zoom function and some headphones; otherwise, the videos disturb other patients.	*I think the combination of boxes and videos was great. I have no suggestions for improvements.*
Q6. I thought there was too much inconsistency in this system.	56 (46.7)	52 (43.3)	8 (6.7)	1 (0.8)	3 (2.5)	Future perspectives on usability and design	
						This was unpersonal… I think it could be more personal. Give me test result and not general information like the videos.	*We loved that when something happens on the screen, then it was also reflected in real life. For example, when the screen said the doctor was on his way—he actually came.*

Q7. I would imagine that most people would learn to use this system very quickly.	0 (0.0)	0 (0.0)	15 (12.5)	53 (44.2)	52 (43.3)	Family implications	
						Many elderly would not be able to use this or if they are sick. The system needs to be simplified for the elderly.	*My mother is not able to remember what she is planned for today. I think it was great for me to see she is waiting for X rays.*
Q8. I found the system very cumbersome to use.	70 (58.3)	42 (35.0)	5 (4.2)	2 (1.7)	1 (0.8)	Future perspectives on usability and design	
						This is new technology, that is often difficult for me.	*I can hear many things happen in the hallway. The systems help me to believe I am not forgotten in this hectic department.*
Q9. I felt very confident using the system.	2 (1.7)	6 (5.0)	4 (3.3)	47 (39.2)	61 (50.8)	Family implications	
						I did not use it much. My family always help me with my phone and so… If they were here, it would maybe have been more meaningful.	*It was nice for me to have a system that told us when things were going to happen. My wife fell asleep, and I knew I did not need to wake her up before I could see the box turned into the blue color. It was easy to understand.*
Q10. I needed to learn a lot of things before I could get going with this system.	67 (55.8)	42 (35.0)	7 (5.8)	4 (3.3)	0 (0.0)	Future perspectives on usability and design	
						You should consider when this system is available… Many things happen in the begging of the stay. This is just one more thing…. However, the intention of the system is good.	*I have been in the hospital many times before, so I knew what was going to happen.*
Q11. I think the system provided a great overview of my stay.	0 (0.0)	2 (1.7)	10 (8.3)	39 (32.5)	69 (57.5)	Means towards empowerment	
						The system gave me nothing useful. The information displayed could be figured out without the system.	*You got the hang of something good here. The system made me calm… I have never met such a system before. Nevertheless, I now realize I needed it.*
Q12. The information in the system made sense to me.	1 (0.8)	5 (4.2)	9 (7.5)	45 (37.5)	60 (50.0)	Means towards empowerment	
						Some of the videos were too long. However, I really liked those which were short and precise.	*I think it all made sense to me. The information in the videos were useful, and because it was provided by sound and pictures it was easy to adapt.*

Table 4: Joint display showing quantitative and qualitative data.
