# Supplementary material for: Usability and Evaluation of a Health Information System in the Emergency Department: Mixed Methods Study
Source: JMIR Hum Factors. 2024 Feb 21;11:e48445. doi: 10.2196/48445 (PMC10918535; doi:10.2196/48445)
Supplement: Multimedia Appendix 2 [file humanfactors_v11i1e48445_app2.pdf]

Supplementary files for the method section.

Method

As the System Usability Scale (SUS) has been previously translated and validated in a Danish hospital context,[1] it was considered suitable for use in this study. However, no cognitive debriefings or adjustments were found from an emergency department (ED), and thus the authors found it necessary to ensure that the questions were relevant and understandable for the population in the emergency department. The process is reported in the following. When translating and validating questionnaires, the principles of good practice for the translation and cultural adaptation process for patient-reported outcomes (PRO) guidelines by ISPOR should be followed.[2] As the SUS has already been translated and validated in Danish, we focused on its understandability and adapting the questions to an ED population, seeking to determine whether any modification of the questions was needed. Therefore, we addressed phase 7, “cognitive debriefing,” of the eight phases recommended by the ISPOR guidelines.[2]

Recruitment and data collection:

We sampled respondents for the cognitive debriefing through purposive sampling. In this way, we aimed to deliberately maximize the sample variation in terms of age and gender, aligned with the ED population in general. The first author (CØ) was present in the ED, and together with the nurse coordinator she identified eligible patients. The nurse coordinator asked the patients if they were interest in participating, and if they agreed CØ discussed the questions with them using the “thinking aloud” method. Notes were taken during each interview.
All patients received verbal and written information about the study and provided written consent to participate.

Results:

Six patients agreed to participate, and one declined due to a lack of interest.

Participant	Gender	Age
**1**	Female	88 years old
**2**	Female	57 years old
**3**	Male	77 years old
**4**	Male	80 years old
**5**	Female	33 years old
**6**	Male	60 years old

Table 1: Patient characteristics.

Three questions were considered difficult to understand (please find final changes in Table 2):
Q1: The word *frequently* did not make sense, as the word indicated further interactions with the ED. Patients in the ED did not expect to come to the ED often, as no follow-up was planned in that department.
Q4: All patients stated that it would have been easier to understand the question if it said *help from the staff instead of a technical person*; therefore, we changed the word.
Q5: The word *integrated* was a difficult word to understand thus, it was changed to a plainer language.

**System Usability Scale statement**	**Modified statement in Danish**	**Statement in Danish**
1. I think that I would like to use this system frequently.	I think I would like to use this system, if I am admitted again.	Jeg tror godt, at jeg kunne tænke mig at bruge systemet, hvis jeg kommer på hospitalet igen.
2. I found the system unnecessarily complex.	I found the system unnecessarily complex.	Jeg synes, at systemet var unødigt kompliceret at bruge.
3. I thought the system was easy to use.	I thought the system was easy to use.	Jeg synes, at systemet var nemt at bruge.
4. I think that I would need the support of a technical person to be able to use this system.	I think that I would need help from the staff to be able to use this system.	Jeg tror, at jeg ville få behov for hjælp fra personalet for at kunne bruge systemet.
5. I found the various functions in this system to be well integrated.	I found the various functions in the system to be well correlated.	Jeg synes, at mange af systemets funktioner hang godt sammen med hinanden.
6. I thought there was too much inconsistency in this system.	I thought there was too much inconsistency in this system.	Jeg synes, at systemet var usammenhængende.
7. I would imagine that most people would learn to use this system very quickly.	I would imagine that most people would learn to use this system very quickly.	Jeg tror, at de fleste hurtigt kan lære at bruge dette system.
8. I found the system very cumbersome to use.	I found the system very cumbersome to use.	Jeg synes, at systemet var meget besværligt at bruge.
9. I felt very confident using the system.	I felt very confident using the system.	Jeg følte mig sikker på, at jeg kunne bruge systemet.
10. I needed to learn a lot of things before I could get going with this system.	I needed to learn a lot things before I could get going with this system.	Jeg var nødt til at lære en masse ting, for at komme i gang med at bruge systemet.

Table 2: The System Usability Scale and our modifications.

References:

1. Hvidt JCS, Christensen LF, Sibbersen C, et al. Translation and validation of the system usability scale in a Danish mental health setting using digital technologies in treatment interventions. *International Journal of Human–Computer Interaction* 2020; 36: 709-716.
2. Wild D, Grove A, Martin M, et al. Principles of Good Practice for the Translation and Cultural Adaptation Process for Patient-Reported Outcomes (PRO) Measures: report of the ISPOR Task Force for Translation and Cultural Adaptation. *Value Health* 2005; 8: 94-104. 2005/04/05. DOI: 10.1111/j.1524-4733.2005.04054.x.
